# Supplementary material for: Efficacy and safety of semaglutide for obesity and hyperphagia in adults with Prader-Willi syndrome
Source: Front Endocrinol (Lausanne). 2026 Sep 3;17:1881392. doi: 10.3389/fendo.2026.1881392 (PMC13581520; doi:10.3389/fendo.2026.1881392)
Supplement: Supplementary file 1 [file Supplementaryfile1.pdf]

Efficacy and safety of semaglutide for obesity and hyperphagia in adults with Prader-Willi syndrome

Shahd Ahmed [1], Nicola Bridges [2], Anthony P. Goldstone [1,3*]

Affiliations:

[1] PsychoNeuroEndocrinology Research Group, Division of Psychiatry, Department of Brain Sciences, Faculty of Medicine, Imperial College London, Hammersmith Hospital, London UK

[2] Department of Paediatric Endocrinology, Chelsea and Westminster Hospital, London UK

[3] Department of Endocrinology, Imperial College Healthcare NHS Trust, Hammersmith Hospital, London UK

* Corresponding author: Dr. Tony Goldstone, Room 2S5B, 2nd Floor Commonwealth Building, Division of Psychiatry, Department of Brain Sciences, Imperial College London, Hammersmith Hospital, Du Cane Road, London W12 0NN, UK. E-mail: tony.goldstone@imperial.ac.uk. Telephone: +44 20 7594 5989. ORCID: 0000-0001-8179-7071.

Journal: Frontiers in Endocrinology

SUPPLEMENTARY MATERIAL

Supplementary Table 1. Summary of STEP clinical trials of semaglutide for overweight/obesity.

Supplementary Table 2. Summary of changes in outcomes prior to and after starting semaglutide.

Supplementary Table 3. Tc-99 gastric emptying findings in adults with PWS and type 2 diabetes mellitus.

Questions from the Hyperphagia Questionnaire for Clinical Trials (HQ-CT)

Trial	Author	Year	Population BMI status	Treatment or withdrawal	T2DM	Age group	% T2DM	Other Rx	Group	N start	N end	Dropout %	Age (y), mean ± SD	Maximum dose (mg)	Duration (weeks)	Start weight	BMI baseline (kg/m²)	Δ mean weight (kg)	mean diff kg vs. placebo	P	Δ mean % weight	mean diff % wt loss vs. placebo	P	% ≥5% weight loss	% ≥10% weight loss	% ≥15% weight loss	% ≥20% weight loss
STEP 1	Wilding	2021	OW/OB	treatment	o	adult	0	lifestyle	Semaglutide	1306	1059	18.9	46 ± 13	2.4	68	105.4 ± 22.1	37.8 ± 6.7	-15.3	-12.7	<0.001	-14.9	-12.5	<0.001	86.4	69.1	50.5	32
									Placebo	655	499	23.8	47 ±12			105.2 ± 21.5	38.0 ± 6.5	-2.6			-2.4			31.5	12	4.9	1.7
STEP 2	Davies	2021	OW/OB	treatment	yes	adult	100	lifestyle	Semaglutide	404	387	4.2	>18	2.4	68	99.9 ± 22.5	35.9 ± 6.4	-9.7	-6.2		-9.6	-6.2	<0.0001	68.8	45.6	25.8	
									Placebo	403	375	6.9	>18			100.5 ± 20.9	35.9 ± 6.5	-3.5			-3.4			28.5	28.7	13.7	
STEP 3	Wadden	2021	OW/OB	treatment	o	adult	0	LCD 8w	Semaglutide	407	373	8.4	46 ± 13	2.4	68	106.9 ± 22.8	38.1 ± 6.7	-16.8	-10.6	<0.001	-16	-10.3	<0.001	86.6	75.3	55.8	35.7
								plus behav therapy	Placebo	204	189	7.4	46 ± 13			103.7 ± 22.9	37.8 ± 6.9	-6.2			-5.7			47.6	27	13.2	3.7
STEP 4	Rubino	2021	OW/OB	withdrawal	o	adult	0	lifestyle	Semaglutide	535	518	3.2	47 ±12	2.4	48	96.5 ± 22.5	34.5 ± 6.9	-7.1	-13.2	<0.001	-7.9	-14.8	<0.001				
									Placebo	268	248	7.5	46 ± 12			95.4 ± 22.7	34.1 ± 7.1	6.1			6.9						
STEP 5	Garvey	2022	OW/OB	treatment 2y	o	adult	0	lifestyle	Semaglutide	152	144	5.3	47 ±12	2.4	104	105.6 ± 20.8	38.6 ± 6.7	-16.1	-12.9		-15.2	-12.6	<0.0001	77.1	61.8	52.1	36.1
							(42-51% preDM)		Placebo	152	128	15.8	47 ±10			106.5 ± 23.1	38.5 ± 7.2	-3.2			-2.6			34.4	13.3	7	2.3
STEP 6	Kadowaki	2022	OW/OB	treatment	some	adult	25	lifestyle	Semaglutide	199	193	3.0	52 ± 12	2.4	68	86.9 ± 16.5	32.0 ± 4.6				-13.2	-11.1	<0.0001	82.9	60.6	40.9	
						(East Asian)			Placebo (pooled)	101	100	1.0	50 ± 9			90.2 ± 15.1	31.9 ± 4.2				-2.1			21	41.8	24.5	
STEP 8	Rubino	2022	OW/OB	treatment	o	adult	0	lifestyle	Semaglutide	126	117	7.1	48 ±14	2.4	68	102.5 ± 25.3	37.0 ± 7.4	-15.3	-13.7		-15.8	-13.9		87.2	70.9	55.6	38.5
									Placebo	85	78	8.2	51 ± 12			103.7 ± 22.5	37.2 ± 6.4	-1.6			-1.9			29.5	15.4	6.4	2.6
STEPTEENS	Weghuber	2022	OB	treatment	few	adolescent	4	lifestyle	Semaglutide	134	131	2.2	15.5 ± 1.5	2.4	68	109.9 ± 25.2	37.7 ± 6.7	-5.8	-5.9		-16.1	-16.7	<0.001	72.5	61.8	53.4	37.4
						12-18y			Placebo	67	62	7.5	15.3 ± 1.6			102.6 ± 22.3	35.7 ± 5.4	0.1			0.6			17.7	8.1	4.8	3.2

Supplementary Table 1. Summary of STEP clinical trials of semaglutide for overweight/obesity.

Abbreviations: BMI, body mass index; o, no; OB, obesity; OW, overweight; preDM, prediabetes; Rx, prescription; SD, standard deviation; T2DM, type 2 diabetes mellitus; y, years. Data is taken from the following clinical trials:

Garvey WT, Batterham RL, Bhatta M, Buscemi S, Christensen LN, Frias JP, Jodar E, Kandler K, Rigas G, Wadden TA, Wharton S, Group SS. Two-year effects of semaglutide in adults with overweight or obesity: the STEP 5 trial. Nat Med. 28:2083-2091, 2022.

Kadowaki T, Isendahl J, Khalid U, Lee SY, Nishida T, Ogawa W, Tobe K, Yamauchi T, Lim S, investigators S. Semaglutide once a week in adults with overweight or obesity, with or without type 2 diabetes in an east Asian population (STEP 6): a randomised, double-blind, double-dummy, placebo-controlled, phase 3a trial. Lancet Diabetes Endocrinol. 10:193-206, 2022.

Rubino D, Abrahamsson N, Davies M, Hesse D, Greenway FL, Jensen C, Lingvay I, Mosenzon O, Rosenstock J, Rubio MA, Rudofsky G, Tadayon S, Wadden TA, Dicker D, Investigators S. Effect of Continued Weekly Subcutaneous Semaglutide vs Placebo on Weight Loss Maintenance in Adults With Overweight or Obesity: The STEP 4 Randomized Clinical Trial. JAMA. 325:1414-1425, 2021.

Rubino DM, Greenway FL, Khalid U, O'Neil PM, Rosenstock J, Sorrig R, Wadden TA, Wizert A, Garvey WT, Investigators S. Effect of Weekly Subcutaneous Semaglutide vs Daily Liraglutide on Body Weight in Adults With Overweight or Obesity Without Diabetes: The STEP 8 Randomized Clinical Trial. JAMA. 327:138-150, 2022.

Wadden TA, Bailey TS, Billings LK, Davies M, Frias JP, Koroleva A, Lingvay I, O'Neil PM, Rubino DM, Skovgaard D, Wallenstein SOR, Garvey WT, Investigators S. Effect of Subcutaneous Semaglutide vs Placebo as an Adjunct to Intensive Behavioral Therapy on Body Weight in Adults With Overweight or Obesity: The STEP 3 Randomized Clinical Trial. JAMA. 325:1403-1413, 2021.

Weghuber D, Barrett T, Barrientos-Perez M, Gies I, Hesse D, Jeppesen OK, Kelly AS, Mastrandrea LD, Sorrig R, Arslanian S, Investigators ST. Once-Weekly Semaglutide in Adolescents with Obesity. N Engl J Med. 387:2245-2257, 2022.

Wilding JPH, Batterham RL, Calanna S, Davies M, Van Gaal LF, Lingvay I, McGowan BM, Rosenstock J, Tran MTD, Wadden TA, Wharton S, Yokote K, Zeuthen N, Kushner RF, Group SS. Once-Weekly Semaglutide in Adults with Overweight or Obesity. N Engl J Med. 384:989-1002, 2021.

Statistics	T2DM	On insulin	Prior follow-up duration	Maximum tolerated dose	Post follow-up duration on semaglutide	Time on maximum dose	Δ Weight prior	Δ Weight post	Δ Weight prior	Δ Weight post	Δ Weight per month prior	Δ Weight per month post	Δ HbA1c prior	Δ HbA1c post	Δ Insulin dose prior	Δ Insulin dose post	Δ HQ-CT total prior	Δ HQ-CT total post	Δ FSZ post	Δ LDL-C prior	Δ LDL-C post	Δ HDL-C prior	Δ HDL-C post	Δ Total / HDL-C prior	Δ Total / HDL-C post	Δ ALT prior	Δ ALT post	Δ AST prior	Δ AST post	Δ FIB-4 prior	Δ FIB-4 post
			(months)	(mg)	(months)	(months)	(kg)	(kg)	(%)	(%)	(%)	(%)	(mmol/mol)	(mmol/mol)	(units)	(units)	(/36)	(/36)	(/80)	(mmol/L)	(mmol/L)	(mmol/L)	(mmol/L)			(IU/L)	(IU/L)	(IU/L)	(IU/L)		
**Weight loss ≤5% in 7mo pre-semaglutide**																															
	yes	o	15.5	(1.0)	33.1	29.4	-0.1	4.1	-0.2	7.4	-0.01	0.22	**2**	**17**	0	0	**-3** [a]	**-14**	?	-0.25	0.19	0.02	-0.19	-0.36	0.71	-95	33	-26	-2	0.10	-0.10
	yes	o	9.2	1.0	11.3	5.3	15.8	8.6	13.2	6.3	1.43	0.56	2 [b]	?	0	0	**0**	**-1**	?	**0.55** [c]	**-0.20**	0.2 [d]	0	-0.30 [e]	-0.20	0 [f]	-1	4 [g]	0	0.08 [h]	0.01
	yes	yes	12.3	(1.7)	36.1	5.7	9.0	0	11.1	0	0.34	0	**37** [i]	**-33**	-8	8	?	?	?	-0.85 [j]	0.16	**-0.51** [k]	**0.12**	0.09 [l]	-0.13	-1 [m]	3	-5 [n]	3	0 [o]	0.01
	yes	yes	7.6	1.7	15.9	10.3	4.0	0.7	3.3	0.6	0.43	0.03	**-34**	**-39**	5	-12	**7**	**-3**	-5	**-0.30**	**0**	0.30	-0.10	-1.70	0.10	**-62**	**-33**	**-72**	**-17** [p]	-0.32	-0.13 [q]
	yes	o	18.5	1.7	20.7	2.6	?	-11.7	?	-10.3	?	-0.51	**43**	**-63** [r]	0	0	?	**-10** [s]	?	?	**0.38** [t]	?	0 [u]	?	0.03 [v]	?	**-21** [w]	?	-1 [x]	?	-0.01 [y]
	yes	yes	12.4	2.4	11.7	2.6	4.5	4.5	4.8	4.7	0.39	0.40	**-39**	**-25**	15	-15	0	-2	-3	-0.12	0.60	**-0.07**	**0**	-0.01	0.70	-9	-2	5	-3	0.05	-0.04
	yes	o	15.6	2.4	16.6	7.2	0.8	-2.2	0.8	-2.1	0.05	-0.12	-2	-4	0	0	-14 [z]	-6	-3	?	?	?	?	?	?	?	?	?	?	?	?
	yes	yes	23.0	2.4	17.7	13.1	12.5	-7.1	12.1	-6.1	0.53	-0.35	**31**	**9** [aa]	20	-22	?	**-3**	-4	0.60	0	**-0.20**	**0.10**	**2.10**	**-1.50**	**4** [ab]	**-29** [ac]	**-8** [ad]	**-18** [ae]	-0.06	0.02 [af]
	yes	o	10.3	2.4	30.6	11.0	-2.4	-11.5	-2.3	-11.3	-0.22	-0.38	0	2	0	0	**2** [ag]	**-10**	-11	**0.99**	**-2.11**	**-0.62**	**0.06**	**4.00**	**-3.37**	**68**	**-61**	**19**	**-19**	0.09	-0.18
	o	o	11.0	1.0	28.2	18.0	5.0	-34.5	4.1	-27.0	0.40	-0.95	-4	-4	n/a	n/a	**-4** [ah]	**-3**	10	**-0.18**	**0**	0.22	0	-0.44	-0.10	7	2	4	3	0.13	0.18
	o	o	9.7	2.4	8.7	2.6	3.4	7.9	2.8	6.5	0.29	0.75	-3	0	n/a	n/a	1 [ai]	-7	-3	-0.20 [aj]	0.30	-0.20 [ak]	0.20	0.20 [al]	0.70	-25	4	-7	2	0.21	-0.11
	o	o	12.4	2.4	12.4	2.7	10.4	-4.8	12.9	-5.3	1.24	-0.42	2 [am]	64	n/a	n/a	**19** [an]	**-21**	5	?	?	?	?	?	?	?	?	?	?	?	?
**mean**			13.1		20.3	9.2	5.7	-3.8	5.7	-3.1	0.44	-0.06	2.9	-6.9	8.0	-10.3	-1.3	-7.3	-1.8	0.03	-0.07	-0.10	0.019	0.40	-0.31	-12.6	-10.5	-9.6	-5.2	0.03	-0.04
**median**			12.4		17.2	6.5	4.5	-1.1	4.1	-1.1	0.39	-0.06	1.0	-4.0	10.0	-13.5	0	-6.0	-3.0	-0.18	0.08	-0.07	0	-0.01	-0.04	-1.0	-1.5	-5.0	-1.5	0.08	-0.03
**SD**			4.4		9.4	8.0	5.6	11.8	5.6	9.9	0.50	0.49	24.9	33.4	12.4	12.9	8.9	6.1	6.4	0.57	0.75	0.32	0.11	1.67	1.26	45.9	26.1	26.4	9.1	0.15	0.10
**P vs. baseline**							**0.007**	0.28	**0.007**	0.31	**0.014**	0.66	0.69	0.51	0.29	0.21	0.66	**0.003**	0.47	0.89	0.78	0.40	0.60	0.50	0.46	0.44	0.23	0.31	0.10	0.56	0.31
**Cohen's d vs. baseline**							**1.02**	-0.32	**1.01**	-0.31	**0.89**	-0.13	0.12	-0.21	**0.65**	**-0.80**	-0.15	**-1.19**	-0.27	0.05	-0.09	-0.30	0.17	0.24	-0.24	-0.27	-0.40	-0.36	**-0.57**	0.20	-0.34
**P post vs. prior**								**0.042**		**0.037**		**0.040**		0.48		0.24		0.10			0.74		0.41		0.47		0.89		0.57		0.26
**Cohen's d post vs. prior**								**-0.70**		**-0.72**		**-0.71**		-0.22		**-0.73**		**-0.61**			-0.12		0.29		-0.25		0.05		0.20		-0.41
**If abnormal values at baseline**																															
**n**													6	6			7	9													
**P vs. baseline**													0.67	0.13			0.33	**0.006**													
**Cohen's d vs. baseline**													0.18	**-0.74**			0.40	**-1.23**													
**P post vs. prior**														0.21				0.06													
**Cohen's d post vs. prior**														**-0.59**				**-0.86**													
**Weight loss >5% in 7mo pre-semaglutide**																															
	yes	yes	14.0	0.5	33.3	32.2	-23.4	-7.3	-23.4	-9.5	-1.67	-0.29	-3	-2	-16	-14	?	-14 [ao]	?	-0.16	0.54	-0.30	0.45	0.68	-0.76	-2	8	3	-5	-0.21	0.47
	yes	o	9.5	(1.0)	5.7	1.8	1.4	13.7	1.2	10.8	0.12	1.90	3	-1	0	0	-4 [ap]	0	?	-0.20	-0.10	0	-0.10	-0.20	0.30	26	-21	19	-9	0.22	-0.08

Supplementary Table 2. Summary of changes in outcomes prior to and after starting semaglutide.

Summary of changes in weight, glycosylated haemoglobin (HbA1c), insulin dose, hyperphagia (HQ-CT), food environment (FSZ), lipid profile, liver enzymes, FIB-4 liver score from prior visit to baseline visit, and baseline to last visit, for all participants (n=14). Participants were split into two groups, those who had ≤5% weight loss (n=12, included in analysis) or >5% weight loss (n=2, excluded from analysis) in the seven months prior to starting semaglutide. Doses in brackets indicate participants still undergoing dose up-titration. Analysis of insulin dose change includes only those on insulin at baseline. For those individuals with abnormal values

at baseline (HbA1c >48mmol/mol, HQ-CT >13/36, LDL-cholesterol >3.0mmol/L, HDL-cholesterol <1.0mmol/L for males and <1.2mmol/L for females, total/HDL-cholesterol ratio >5.0, ALT >34 IU/L, AST >40 IU/L), the Δ post or prior to baseline are given in bold. P values <0.05 and Cohen's d >0.50 are in bold. To convert HbA1c (mmol/mol) to (%) use the formula: % = (0.09148 × mmol/mol) + 2.15. Abbreviations: Δ, change; ?, unknown; ALT, alanine transaminase; AST, aspartate aminotransferase; C, cholesterol; FIB-4, fibrosis-4; FSZ, food safe zone; HbA1c, glycated haemoglobin; HDL, high-density lipoprotein; HQ-CT, hyperphagia questionnaire for clinical trials; LDL, low-density lipoprotein; LLN, lower limit normal; o, no; SD, standard deviation; T2DM, type 2 diabetes mellitus; ULN, upper limit normal.

Superscripts indicate data collected at different time point than overall follow-up duration before/after semaglutide, in months: [a] 44.6, [b] 6.1, [c] 6.1, [d] 6.1, [e] 6.1, [f] 3.0, [g] 3.0, [h] 3.0, [i] 32.6, [j] 32.6, [k] 32.6, [l] 32.6, [m] 32.6, [n] 32.6, [o] 32.6, [p] 9.2, [q] 9.2, [r] 18.2, [s] 18.2, [t] 18.2, [u] 18.2, [v] 18.2, [w] 18.2, [x] 18.2, [y] 18.2, [z] 50.5, [aa] 11.5, [ab] 27.3, [ac] 19.1, [ad] 27.3, [ae] 19.1, [af] 19.1, [ag] 46.2, [ah] 52.7, [ai] 16.1, [aj] 16.1, [ak] 16.1, [al] 16.1, [am] 10.4, [an] 27.1, [ao] 9.4, [ap] 7.1.

Age range (yr)	BMI (kg/m2)	HbA1c (%, mmol/mol)	T2DM Rx	Gastric emptying t1/2 baseline (NR <60 mins)	Gastric emptying t1/2 on Liraglutide (NR <60 mins)	Liraglutide dose (mg)	Clinical decision about GLP-1 analogue
20-24	39.5	9.6%, 81	Met Th I	48	83	1.8	Continue GLP-1RA
20-24	30.7	6.8%, 51	Met	25	49	1.8	Continue GLP-1RA
40-44	41.4	13.3%, 122	Met, I	-	79	1.2	GLP-1RA dose increase CI, started Th
25-29	36.6	107	Met, Th	16	348	1.8	GLP-IRA discontinued
30-34	54	10.2%, 88	?	>180	-	-	GLP-1RA CI
20-24	40.4	9.9%, 85	Met, Th	32	-	-	GLP-1RA not CI
15-19	64.3	FBG 4.9 mmol/L	Met, S	16	-	-	GLP-1RA not CI

Supplementary Table 3. Tc-99 gastric emptying findings in adults with PWS and type 2 diabetes mellitus.

Data from 4 males and 3 females. Abbreviations: BMI, body mass index; CI, contraindicated; FBG, fasting blood glucose; GLP-1RA, GLP-1 receptor agonist; I, Insulin; Met, metformin; mins, minutes; NR, normal range; Rx, treatment; S, sulphonylurea; T2DM, type 2 diabetes mellitus; Th, Thiazolidinedione; t1/2, half-life; yr, year. Adapted from: Ali SN, Bridges N, Al-Nahhas A, Papadopoulou D, Sampson M, Goldstone AP. Assessing gastric emptying before and after Introduction of GLP-1 agonists for glycaemic and body weight control in Prader-Willi Syndrome. Abstracts 31st Annual Meeting of the Obesity Society, Atlanta USA, 2013.

Questions from the Hyperphagia Questionnaire for Clinical Trials (HQ-CT)

Instructions: The following items refer to the person in your care and assessment of his/her food-related behaviour during the past 2 weeks.

(1) During the past 2 weeks, how upset did the person generally become when denied a desired food?
___ Not at all upset
___ A little upset
___ Moderately upset
___ Very upset
___ Extremely upset

(2) During the past 2 weeks, how often did the person try to bargain or manipulate to get more food at meals?
___ Never
___ Up to 2 times a week
___ 3 to 6 times a week
___ Every day
___ Several times a day

(3) During the past 2 weeks, how often did the person forage through trash for food?
___ Never
___ 1 time
___ 2 times
___ 3 times
___ 4 or more times

(4) During the past 2 weeks, how often did the person get up at night to food seek?
___ Never
___ 1 time
___ 2 times
___ 3 times
___ 4 or more times

(5) During the past 2 weeks, how persistent was the person in asking or looking for food after being told “no” or “no more”?
___ Not at all persistent
___ A little persistent
___ Moderately persistent
___ Very persistent
___ Extremely persistent

(6) During the past 2 weeks, outside of normal meal times, how much time did the person generally spend asking or talking about food?
___ Less than 5 minutes a day
___ 5 to 15 minutes a day
___ 15 to 30 minutes a day
___ 30 minutes to 1 hour a day
___ More than 1 hour a day

(7) During the past 2 weeks, how often did the person try to sneak or steal food (that you are aware of)?
___ Never
___ 1 time
___ 2 times
___ 3 times
___ 4 or more times

(8) During the past 2 weeks, when others tried to stop the person from asking about food, how distressed did he or she generally appear?
___ Not at all distressed
___ A little distressed
___ Moderately distressed
___ Very distressed
___ Extremely distressed

(9) During the past 2 weeks, how often did food-related behavior interfere with the person's normal daily activities, such as self-care, recreation, school, or work?
___ Never
___ Up to 2 times a week
___ 3 to 6 times a week
___ Every day
___ Several times a day

Each question is scored 0-4.
HQ-CT total: questions 1-9 (score 0-36)
HQ-CT behaviour: questions 2, 3, 4 and 7 (score 0-16)
HQ-CT drive: questions 1, 5 and 8 (score 0-12)
HQ-CT severity: questions 6 and 9 (score 0-8)

Taken from: Matesevac L, Vrana-Diaz CJ, Bohonowych JE, Schwartz L, Strong TV. Analysis of Hyperphagia Questionnaire for Clinical Trials (HQ-CT) scores in typically developing individuals and those with Prader-Willi syndrome. *Sci Rep*. 13:20573, 2023.

Contact information for permission to use the HQ-CT: Foundation for Prader-Willi Research (www.fpwr.org), info@fpwr.org.
